# Social approaches to COVID-19 pandemic response: effectiveness and practicality in sub-Saharan Africa

**DOI:** 10.11604/pamj.supp.2020.37.1.25183

**Published:** 2020-09-02

**Authors:** Uchenna Anderson Amaechi, Babasola Olufemi Sodipo, Chukwudi Arnest Nnaji, Ayomide Owoyemi, Kasarachi Omitiran, Ijeoma Nkem Okedo-Alex, Ejemai Eboreime, Olufemi Ajumobi

**Affiliations:** 1 Strategy, Investment and Impact Division, Global Fund to Fight AIDS, Tuberculosis and Malaria, Geneva, Switzerland,; 2 Harvard T.H. Chan School of Public Health, Boston, United States of America,; 3 School of Public Health and Family Medicine, University of Cape Town, Cape Town, South Africa,; 4 Cochrane South Africa, South African Medical Research Council, Cape Town, South Africa,; 5 Department of Biomedical and Health Information Sciences, University of Illinois, Chicago, United States of America,; 6 Lumiere Health Research Consulting, Abuja, Nigeria,; 7 National Primary Health Care Development Agency (NPHCDA), Abuja, Nigeria,; 8 Department of Community Medicine, Alex Ekwueme Federal University Teaching Hospital Abakaliki, Nigeria,; 9 African Institute for Health Policy and Health Systems, Ebonyi State University, Abakaliki, Nigeria,; 10 Department of Psychiatry, Faculty of Medicine and Dentistry, University of Alberta, Edmonton, Canada,; 11 School of Community Health Sciences, University of Nevada, Reno, Nevada, United States of America

**Keywords:** Social distancing, non-pharmaceutical intervention, school, workplace, mass gathering, community mitigation

## Abstract

**Introduction:**

the threat of the coronavirus disease 2019 (COVID-19) pandemic to health systems and communities in sub-Saharan Africa (SSA) is enormous. Social approaches such as distancing measures are essential components of the public health response to respiratory-related infectious disease outbreaks. Due to socio-economic and broader peculiarities of SSA countries, social approaches that were effective elsewhere may have limited practicality in these contexts, and if practical; may yield different or even adverse results. We highlighted the effectiveness of these social approaches and their practicality in SSA.

**Methods:**

we conducted a comprehensive literature search through multiple databases, to identify articles relevant to social distancing. Findings were thematically summarized.

**Results:**

our review found emerging and varying empirical evidence on the effectiveness of social approaches in the control and mitigation of the COVID-19 pandemic; thus, limiting its applicability in SSA contexts. Nonetheless, our review demonstrates that the effectiveness and practicality of social approaches in SSA contexts will depend on available resources; timing, duration, and intensity of the intervention; and compliance. Weak political coordination, anti-science sentiments, distrust of political leaders and limited implementation of legal frameworks can also affect practicality.

**Conclusion:**

to overcome these challenges, tailoring and adaptation of these measures to different but unique contexts for maximum effectiveness, and investment in social insurance mechanisms, are vital.

## Introduction

The World Health Organization (WHO) declared the coronavirus disease 2019 (COVID-19) outbreak a pandemic on the 11^th^ of March 2020. The pandemic has since spread to more than 192 countries and territories around the world. Evidence shows that COVID-19 is readily transmissible from person to person through air droplets and aerosols [1]. Globally, as governments grapple with the human, social and economic costs of the pandemic, many countries have implemented varying public health measures to stem the tide and flatten the curve of the disease transmission. However, controlling the pandemic is challenging as there is limited evidence on the safety and effectiveness of appropriate medicines and currently no approved vaccines to prevent COVID-19. As a result, countries have adopted social distancing, or more broadly, social approaches to control the pandemic. The Centers for Disease Control and Prevention (CDC) describes social distancing as remaining out of congregate settings, avoiding mass gatherings, and maintaining distance (approximately six feet or two meters) from others when possible [2,3]. The goals of social distancing are to delay the introduction of the pandemic virus into a population; delay the height and peak of infection where there are already cases; and to reduce the total number of infections and hence the total number of severe cases [4] As of 21 July 2020, the pandemic had spread to all African countries (except Western Sahara), with a regional total of over 700,000 cases and about 15000 deaths, while nearly 60 000 new cases and 780 deaths were reported the previous day. Given the current trends in incidence and fatality, coupled with underlying healthcare systems vulnerabilities, it is now thought that region could become the next epicentre of the COVID-19 pandemic. With a youthful population, low rates of urbanization, high rate of poverty, a growing burden of communicable and non-communicable diseases, pervading socioeconomic inequalities and other contextual peculiarities, the pandemic may spread and affect sub-Saharan Africa (SSA) in ways different from the rest of the world [5]. Due to these socioeconomic and broader peculiarities, social approaches that were effective elsewhere may have limited practicality in SSA, and if practical; may yield different or even adverse results. Therefore, the aim of this review was 2-pronged: first, to review and summarize the current evidence on the effectiveness of social approaches to COVID-19 and respiratory disease outbreak response; secondly, to contextualize the findings and practicality of these approaches within SSA.

## Methods

In the first prong, we reviewed the evidence on the effectiveness of social approaches to COVID-19 and respiratory disease outbreak response. To do this, we conducted a literature search of the following electronic databases: MEDLINE (via LitCOVID in PubMed), Google scholar, Cochrane Library, African Index Medicus, and EBSCOhost. To be eligible for inclusion, publications had to assess the effectiveness of social measures used in the response to COVID-19 or other respiratory disease outbreaks in SSA or other settings. Reference lists of relevant articles and documents on databases of the World Health Organization (WHO) and Africa Centres for Disease Control and Prevention (Africa CDC) were also scanned for potentially relevant articles. See Table 1 for the PubMed/MEDLINE search strategy, which was adapted for other databases using appropriate search terms and Boolean operators. In the second prong, we contextualized the identified evidence by discussing the practical implication of the approaches within SSA contexts for this, we searched both published and grey literature (including the news media) specific to SSA to identify relevant publications for contextualizing our findings from the first prong. Searches were conducted between 22 April and 20 July 2020. Findings were summarized narratively and reported under pre-specified themes. We have adapted the categorization of Non-Pharmacological Interventions (NPIs) proposed by Fong *et al*. [6], to thematically conceptualize and categorize the social approaches to COVID-19 response discussed in this review. The categorization by Fong *et al*. is comprehensive, specific to respiratory disease pandemics and is widely cited across literature sources and publications addressing NPIs, hence highly relevant to our review. Further, the rationale for using this framework was due to its clear definition of each social approach. Based on that, we categorized the social approaches into five distinct but interrelated themes: 1) Isolation and quarantine; 2) Closure of schools; 3) Closure of workplaces; 4) Avoiding/cancellation of mass gathering; 5) Restriction of movement. We highlight in each category the evidence on the rationale and effectiveness of the interventions as well as their contextual implications within SSA.

**Table 1:** PubMed/MEDLINE search strategy

Search#	Search terms
#1	(COVID-19 [Title/Abstract] OR corona [Title/Abstract] OR 2019-nCoV [Title/Abstract] OR “Coronavirus disease” [Title/Abstract] OR SARS-CoV-2 [Title/Abstract] OR “Severe acute respiratory syndrome coronavirus 2” [Title/Abstract]
#2	Pandemic[Title/Abstract] OR outbreak [Title/Abstract] OR epidemic[Title/Abstract]
#3	#1 AND #2
#4	Isolation[Title/Abstract] OR quarantine[Title/Abstract] OR “Social distancing” [Title/Abstract] OR “physical distancing” [Title/Abstract] OR lockdown[Title/Abstract] OR “school closure” [Title/Abstract] OR “school dismissal” [Title/Abstract] OR “workplace closure” [Title/Abstract] OR teleworking[Title/Abstract] OR “mass gathering” [Title/Abstract] OR “public gathering” [Title/Abstract] OR “movement restriction”[Title/Abstract]
#5	Africa OR “sub-Saharan Africa” OR “Africa South of the Sahara”
#6	#3 AND #4 AND #5

## Results

**Isolation and quarantine:** the severe acute respiratory syndrome coronavirus 2 (SARS-CoV-2) spreads primarily by human to human transmission through infected droplets [7,8] .Hence, it is reasonable to institute measures such as the removal of symptomatic individuals from the general population (isolation) and those who have had contact with an infected individual but are not presently displaying symptoms (quarantine) to control the spread of the disease [9]. Empirical evidence on the effectiveness of isolation and quarantine in tackling disease outbreaks is mixed, particularly for respiratory diseases 8. Generally, the effectiveness of these measures depends on the characteristics of the pathogen, its symptomatology and transmission dynamics [9]. Commonly, studies concluded that respiratory disease outbreaks are likely to be effectively contained by case isolation in the absence of quarantine of contacts, only if isolation is implemented stringently and effectively [10-12]. However, the number of infections averted through isolation alone decreases with the interval between onset of symptoms and the time of isolation, and with a higher basic reproduction number (Ro). Given the high variability of the Ro of the novel coronavirus; the substantial proportion of asymptomatic cases which may delay diagnosis and subsequent isolation´ and the potential of asymptomatic transmission, isolation alone is not likely to be sufficient for mitigating the spread. Therefore, a combination of isolation and quarantine is warranted.

**Practicality of isolation and quarantine in sub-Saharan Africa:** the potential health, social, and economic impacts of the spread of COVID-19 in Africa are important considerations for governments and public health authorities in SSA to take pragmatic steps towards stemming the tide of transmission. While isolating cases and quarantining those exposed are reasonable interventions that are potentially effective for controlling infectious disease outbreaks, they are resource-intensive [12]. Given the substantial evidence on their effectiveness, both measures may offer a ray of hope for countries and communities in SSA, if appropriately implemented in combination with optimal testing capacity, as well as prompt and efficient contact tracing. Many African countries have invested in isolation facilities in an effort to better enforce the isolation of confirmed cases for treatment and monitoring, which may not be feasible with self-isolation [13]. As COVID-19 continues to surge while movement restrictions are being eased across SSA countries, there is a need for more isolation facilities to meet the increasing demand from the rising number of cases. Importantly in SSA contexts, there is a need for 'isolation' facilities for not only confirmed cases, but also contacts of confirmed cases and those with significant exposure such as history of travel to transmission hotspots. This is because, these persons may have been infected, but remain asymptomatic and without access to immediate testing due to limited testing capacity - a trend that is typical across SSA currently. Where testing is not immediately feasible, such persons can be relocated to designated isolation/quarantine facilities and monitored until testing is possible, or for an appropriate number of days (usually up to 14 days of remaining symptom free) [13,14].

Isolation/quarantine facilities may also be crucial for contacts residing in overcrowded housing conditions and those who are homeless. Due to overcrowded homes, particularly in urban slums and informal settlements, implementing self-isolation/quarantine may expose other persons in the household and may lead to further community transmission. In addition, these living conditions may make staying indoors and complying with isolation/quarantine for the appropriate duration unbearably uncomfortable, particularly on hot days. Furthermore, the lack of improved water supply and sanitation facilities in communities may also complicate self-isolation of cases and voluntary quarantine of contacts of cases. As such, designated isolation/quarantine facilities could be an avenue for dealing with these practical and contextual constraints. Moreover, seven out of ten Africans work in the informal sector with implications for the practicality of self-isolation/quarantine. There is a high likelihood of non-compliance with these self-isolations /quarantine if people are faced with the risks of income or job losses while absent from work [15]. Informal occupational settings make it difficult for jobs and incomes to be guaranteed in such cases. These challenges call for sector-agnostic policies and legislative frameworks that guarantee the payment of employees by their employers, while temporarily absent from work due isolation/quarantine. As new cases peak, government-designated isolation or quarantine facilities may become overwhelmed and insufficient to hold new cases [16]. There may be a need for incentivizing informal sector employers to comply with such mandates, such as through tax and loan repayment holidays.

While the limitation of rights and freedom of movement during the enforcement of isolation and quarantine may be justifiable in the interest of public health in pandemic settings, it carries the inherent risk of being abused and implemented in draconian ways; particularly in the contexts of authoritarian leadership. This would be the case if there is fear, intimidation, abuse, or victimization. Such adverse outcomes have been highlighted by numerous reports of police and military brutality, intimidation and harassment across a number of African countries during the enforcement of movement restrictions. Hence, it is important for isolation and quarantine to be enforced with fairness and within human rights standards [17]. Lastly, the stigmatization of COVID-19 infection, which may hinder willingness to accept positive test results and agree to be isolated, need to be adequately addressed through proper messaging and public health education of communities.

**Workplace closure:** evidence suggests that there is a substantial risk of respiratory disease transmission in occupational settings [18]. Implementation of workplace distancing included scenarios where 50% of the workforce embarked on teleworking for 2 weeks [18,19]. A 23% reduction in the cumulative attack rate (AR) of the 2009 influenza A (H1N1) was reported to have occurred when workplace social distancing was implemented early, in combination with other non-pharmaceutical interventions (NPI), at higher compliance and lower Ro [19,20]. In addition, a reduction in overall and peak AR and delay in epidemic peak occurred was achieved with workplace physical distancing [19]. The effectiveness depends on the intensity of closure, with a 33% workplace closure reported to reduce the AR to <5% and delayed the peak by one week, compared with a 10% workplace closure with less impact [21]. Comparatively, evidence suggests that workplace closure is likely to have a smaller effect on reducing community transmission than school closure during influenza pandemics [6].

**Practicality of workplace closures in SSA:** the majority of the population in SSA lives in poverty and in rural households without social insurance policy. As discussed earlier, the informal sector makes up about 70% of the workforce, and there are many people who rely on a daily income to eke a livelihood [15]. Without social protection, prolonged absence from work portends job losses and food insecurity [22] which is the case in many SSA contexts currently. These altogether make implementing workplace closure early in the pandemic difficult and unsustainable [23]. Formal structures and databases are often lacking to inform equitable distribution of palliatives even if this is considered. If implemented without safety nets, workplace closure can aggravate the socioeconomic impacts of the pandemic, while widening income gaps and inequality. Realistically, the young productive workforce should remain active while the vulnerable elderly and those with co-morbidities are sheltered-in place [24].

**School dismissal and closure:** school dismissal refers to schools remaining open to only administrative and teaching staff, vulnerable children, children of healthcare and essential workers [19]. On the other hand, everyone is denied access during school closure [21]. These terms, though distinct, are used interchangeably. The duration of school closure depends on the timing of its initiation during the pandemic [25]. School closure delayed the epidemic peak by a week or two, reduced influenza transmission by 1 - 50% and up to 90-100% where this is by contact [26]. In Singapore, school closure reduced transmission by 53% [27]. Furthermore, it delayed the peak AR by a median of 11 days and resulted in greater reduction in community transmission [28-31]. However, the reduced risk of symptomatic transmission among school-age children makes school closure less appealing than workplace social distancing in the context of COVID-19 [18]. School closures are most effective if introduced earlier in the pandemic, at low transmissibility (Ro <2) and at higher AR in children compared to the adult population [28]. Disentangling relative contribution of effectiveness of school closures for COVID-19 within the range of quarantine and social distancing measures is difficult. Hence, its independent effectiveness in reducing transmissibility is uncertain without accounting for other social distancing measures [32].

**Practicality of school closures in SSA:** school closure results in reduced parental productivity including loss of health-care staff to childcare duties [28-30,32]. The majority of children of low and middle-income households in SSA attend public schools without the financial, digital and human resources to implement virtual learning during COVID-19 pandemic; unlike their colleagues from higher-income households who attend private schools with such facilities [22]. Most live in houses which are not conducive for studying and lack access to television to avail them of any form of learning. Furthermore, high cost of, and unreliable internet services; lack of computers and teachers competent in digital tools, and erratic electric power supply constitute barriers to virtual learning. While targeted educational approaches like radio or television-based learning could reduce this gap; such appliances are lacking in most rural households. During the 2014 Ebola outbreak, virtual learning was commonly through broadcasting, particularly with educational radio programmes which were aired in Guinea, Liberia and Sierra Leone [33]. While such learning platforms demonstrated some impacts at reducing transmission of the virus, a large proportion of the children did not engage in any form of home-schooling during the outbreak [34]. It has been shown that only 40% of learners benefited in virtual schooling in Liberia, while only 30% did in Sierra Leone [35]. These can lead to lower educational attainment, while widening learning gaps and worsening pervading socioeconomic disparities [22]. Though hybrid learning (in-person and distance learning) is being implemented in South-Africa, context-specific and locally grounded harm-reduction measures such as reduced class size, less contact hours and NPI may be more realistic in SSA [36-38].

**Avoiding/cancellation of mass gatherings:** mass gathering is a planned or spontaneous event where the number of people attending could strain the planning and response resources of the community or country hosting the event [39]. Examples include The Olympic games, Hajj, major sporting, religious events, political campaign rally, “open market” and cultural events [39]. Avoiding mass gatherings seeks to limit the rapid spread of the virus by increasing physical distance or reducing frequency of congregation in socially dense community settings, such as places of worship and sports arena [16]. It involves timely bans on public gatherings and closure. The evidence for avoiding crowding or mass gathering on limiting the spread of COVID-19 is emerging [40,41]. Evidence from observational studies suggests that timely bans on public gatherings and closure of public places, including theaters and churches, helped in reducing the excess death rate during the 1918 pandemic in the United States [42,43]. Evidence also points to a higher attack rate of diseases like influenza in crowded settings; such as during an influenza outbreak that occurred during World Youth Day in 2008 [44]. During the 1918 influenza pandemic, timely ban of public gathering in St Louis were shown to be effective in reducing the spread of the virus and case fatality rates in contrast to Philadelphia that did not implement measures to prohibit mass gatherings [45].The timing of restrictions on mass gatherings closer to epidemic peak may be more effective than restrictions applied much earlier. Mass gatherings are not homogenous, and risk should be assessed on a case-by-case basis [46].

**Practicality of avoidance /cancellation of mass gathering in sub-Saharan Africa:** in many SSA countries, avoiding/restricting mass gathering has been used as a social distancing measure in limiting the spread of the SARS-CoV-2 .There have been reports of compliance in the shutting down of sporting events, in contrast to large pockets of partial to non-compliance with regards to some cultural and religious practices/events like burials, coronation events, weddings, church/mosque services (Tanzania, Ethiopia, Nigeria) etc. In West Africa, there have been negative reactions, including riots, to the closure of places of worship (Senegal) and incidences of 'super spreading' events at evangelical church sessions (in Burkina Faso and South Africa) [47-49]. These pockets of non-compliance in some countries are particularly driven by mixed messages in risk communication or a lack of it from some religious and political leaders, and mistrust in government in some African countries [50]. However, in many cases, religious leaders have encouraged their congregations to stay home and observe distancing measures, while broadcasting their sermons via television, radio and the internet [51]. Again, the implementation of measures to avoid crowding might require a large amount of resources (e.g., financial and trained personnel), surveillance, which might be less feasible in some areas/cities in low-income and middle-income countries in sub-Saharan African countries [20].

**Restriction of movement:** lockdowns and curfews are categorized as restriction/halting of movement with the rationale of ensuring social distancing measures to delay the peak of the infection. While there is currently no consistent definition for lockdowns, we assume a rather more contextual definition to accommodate the variability in implementation. It ranges from hard or full lockdowns (active coercion with the agency of the police/army (state) in maintaining sit- at- home orders and enforcing no entry/exits into territories), to partial lockdowns (less severe restricted movement of people, or curfews -a term that usually refers to a directive from the government to keep people off the streets for a few pre-decided hours, and closure of non-essential work). The lockdown seen in Wuhan, China is the prototype of hard lockdowns, while the one instituted in London is an example of partial lockdowns [52,53]. The point on the type of lockdown is particularly noteworthy as effectiveness depends on the intensity and duration of lockdowns. We found emerging evidence that where they have been instituted, lockdowns may have contributed to a delay in incident cases, fewer cases, hospitalizations, and deaths [54-58].

Ghosal *et al*. carried out a hierarchical cluster analysis to assess the impact of complete lockdown on total infection and death rates across 12 countries that declared lockdown [54]. Data from a 4-week period before and after the announcement of lockdowns revealed an exponential and significant (R2:0.995) decrease in total infection across all the countries [55]. There was a similar decrease in deaths, but this was not universal as New Zealand showed a trend towards an increase in death rates between week two and three post lockdown. Fang *et al*. reported that had a strict lockdown not been instituted in Wuhan-China, COVID-19 cases would have been 65% higher in the 347 Chinese cities outside Hubei province, and 53% higher in the 16 non-Wuhan cities inside Hubei [56],. Similarly, Gatto *et al*. modelling estimates by placing restrictions reduced COVID-19 transmission by a median 45% (42 to 49%) in Italy with over 200 000 hospitalizations averted as of March 25, 2020 [57]. Institution of lockdown measures in the UK resulted in 74% reduction in the average daily number of contacts observed per respondent and reduced R0 from 2.6 pre-lockdown to 0.62 post- lockdown [58].

**Practicality of restriction of movement:** while effective in combating COVID-19, the WHO recognizes restricting movement as a blunt tool with social and economic costs [59]. The practicality of sustaining such measures will, therefore, depend on the degree to which governments are willing and able to put measures in place to mitigate these costs. Nonetheless, governments can likely withstand the costs of a lockdown for only a short period. As a result, it will be important to identify earlier, the goals to be achieved during lockdowns and the corresponding sound strategies. As already identified with workplace closure, a plan to financially support citizens during lockdowns must be put in place to ensure adherence. Providing support is especially important in the context of sub-Saharan Africa where large swathes of the population are in subsistence living, and workers earn daily wages.

## Discussion

Overall, our review suggests emerging and varying empirical evidence on the effectiveness of social approaches such as isolation, quarantine, school and workplace closures, movement restrictions and prohibition of mass gatherings, in the control and mitigation of the COVID-19 pandemic. We highlighted in each category of the social approaches the rationale for implementing the interventions and their contextual implications. To illustrate, while measures such as work, and school closures may be important for reducing the spread of COVID-19. In many SSA social contexts, these may have little practicality due to low internet penetrations to support online learning and teleworking. Similarly, contextual realities such as overcrowded living conditions and water supply deficits may constrain the practicality of the social distancing rule of staying or living at least 2 meters apart and the NPI of regular handwashing. Other challenges in the region include infrastructural deficits, limited technical capacity to undertake contact tracing and surveillance activities, inadequate or lack of social safety nets, weak legal and legislative frameworks, and mixed messages from some political leaders resulting in anti-science sentiments, and a general mistrust and low confidence in political leaders.

Our review is not without its limitations. Given the very broad nature of the topic and time constraints, we were unable to conduct a systematic search of the literature. However, we minimized the risks of literature selection bias by an internal peer-review process that allowed each team member to critically appraise the literature identified by team mates. Because ours was a non-systematic review, it did not require a systematic quality assessment of included literature. As such, our quality assessment is narrative. The sources of the evidence we included in the review were mostly observational and simulation studies, thereby limiting the certainty of the evidence. Further, the certainty of this review´s evidence is limited by the ongoing and rapidly evolving nature of the pandemic, particularly with the lingering uncertainties around transmission dynamics. In addition, the integrated implementation of these social measures makes it difficult to distinguish the specific impact of each intervention from the cumulative effects of combined efforts. Nonetheless, our review demonstrates that social approaches have varying effectiveness and practicality, depending on available resources, the timing of the intervention, duration, intensity, combination with other measures, implementation monitoring, and compliance. For instance, there is substantial evidence that isolation, used together with contact tracing, quarantine and optimal testing, is beneficial in mitigating the spread of the virus [9].

Finally, at the heart of the limited practicality of these social approaches in SSA countries are (1) fundamental challenges in equity: are the measures equitably implemented?, (2) sustainability: can the measures be implemented sustainably?; (3) financial and technical investments: do SSA countries have the economic and technical resources to implement some of these measures; are these economic resources judiciously and efficiently managed and used as planned? and (4) trust: do the citizens trust the messages from their leaders?

## Conclusion

In sum, there is emerging and varying empirical evidence on the effectiveness of the use of selected social approaches in the control of the COVID-19 pandemic in SSA contexts. There is limited practicality in implementing these social approaches due to infrastructural and economic deficits, limited technical capacity, anti-science sentiments, weak political coordination, distrust of political leaders and limited implementation of legal frameworks. To overcome these practical challenges, tailoring and adaptation of these measures to similar but unique contexts for maximum effectiveness, and investment in social insurance mechanisms, are vital.

### What is known about this topic

- The coronavirus disease 2019 (COVID-19) pandemic has spread rapidly across most countries of the world;
- Social approaches such as distancing measures are essential components of the public health response to respiratory-related infectious disease outbreaks;
- Controlling the pandemic is challenging as there is limited evidence on the safety and effectiveness of appropriate medicines, with vaccines currently available for prevention.

### What this study adds

- This review demonstrates varying effectiveness of social approaches such as isolation, quarantine, school and workplace closures, movement restrictions and prohibition of mass gatherings, in the control and mitigation of the COVID-19 pandemic;
- This review contextualizes the implications of these social approaches in sub-Saharan African contexts, summarizing the evidence in one paper, thus making it easy for readers to access the information;
- This review establishes that the effectiveness and practicality of social approaches in SSA contexts will depend on several important factors. Including the availability of resources; timing of the intervention, effective leadership and community trust.
